# Intra-Palpebral Tuberculin Skin Test and Interferon Gamma Release Assay in Diagnosing Tuberculosis Due to *Mycobacterium caprae* in European Bison (*Bison bonasus*)

**DOI:** 10.3390/pathogens11020260

**Published:** 2022-02-17

**Authors:** Anna Didkowska, Blanka Orłowska, Monika Krajewska-Wędzina, Michał Krzysiak, Małgorzata Bruczyńska, Jan Wiśniewski, Daniel Klich, Wanda Olech, Krzysztof Anusz

**Affiliations:** 1 Department of Food Hygiene and Public Health Protection, Institute of Veterinary Medicine, Warsaw University of Life Sciences (SGGW), Nowoursynowska 159, 02-776 Warsaw, Poland; blanka_orlowska@sggw.edu.pl (B.O.); jan_wisniewski1@sggw.edu.pl (J.W.); krzysztof_anusz@sggw.edu.pl (K.A.); 2 Department of Microbiology, National Veterinary Research Institute, Partyzantów 57, 24-100 Puławy, Poland; kappa2@wp.pl; 3 Institute of Forest Sciences, Faculty of Civil Engineering and Environmental Sciences, Bialystok University of Technology, Wiejska 45 E, 15-351 Białystok, Poland; m.krzysiak@st.pb.edu.pl; 4 Białowieża National Park, Park Pałacowy 11, 17-230 Białowieża, Poland; 5 County Veterinary Inspectorate, Orezna 9, 05-501 Piaseczno, Poland; gosia639@wp.pl; 6 Department of Animal Genetics and Conservation, Institute of Animal Sciences, University of Life Sciences (SGGW), Ciszewskiego 8, 02-786 Warsaw, Poland; daniel_klich@sggw.edu.pl (D.K.); wanda_olech@sggw.edu.pl (W.O.)

**Keywords:** ante-mortem diagnostics, tuberculosis, European bison, interferon gamma release assay (IGRA), intra-palpebral tuberculin skin test, *Mycobacterium caprae*, wildlife

## Abstract

Despite the threat posed by tuberculosis (TB) to the protected European bison (*Bison bonasus*), no validated TB tests exist for this species. This pilot study evaluates two tests based on detecting cellular immunity for this purpose: interferon gamma release assay (IGRA) and tuberculin skin test (TST). Ten animals were subjected to ante-mortem and post-mortem examinations. IGRA was performed using a commercial test, and the comparative TST was performed in the eyelids. The lesions were assessed post-mortem and material was collected for mycobacterial culture. The isolated strains were subjected to genotyping. At post-mortem examination, five out of ten individuals demonstrated both tuberculous lesions and positive culture results (*Mycobacterium caprae*). Compared to the palpebral TST, the findings of the IGRA are easier to interpret when diagnosing tuberculosis in European bison.

## 1. Introduction

While post-mortem examination remains the basis of monitoring infectious diseases in wildlife, the importance of ante-mortem diagnostics in these animals is growing. This growth has been driven by the increasing awareness and understanding of the role of wildlife as spill-over and reservoir hosts of pathogens known to pose a threat to livestock and humans, as well as the growing need to care for endangered species (both free-ranging and captive) and wildlife in general. Two such pathogens are *Mycobacterium caprae* and *Mycobacterium bovis*, the etiological agents of tuberculosis (TB) in mammals. Transmission can occur in free-ranging animals, for example, if they share pastures with infected livestock; it can also occur among captive animals if untested animals are introduced.

While Poland has officially been a tuberculosis-free (OTF) country since 2009, this status is based on the percentage of TB-positive cattle herds and is not affected by the number of cases in other animal species, including wildlife. In Poland, TB has been noted in recent years in alpacas (*Vicugna pacos*) [1], pigs (*Sus domestica*) [2], wolves (*Canis lupus*) [3], wild boar (*Sus scrofa*) [4], roe deer (*Capreolus capreolus*) [5], and American bison (*Bison bison*) [6]; however, the highest number of cases were confirmed in European bison (*Bison bonasus*) [7]. The most commonly isolated species are *M. caprae* in wildlife and *M. bovis* in cattle. In European bison, TB has been microbiologically confirmed in both free-living herds, particularly in the Bieszczady Mountains, southern Poland, and in enclosures (ex situ breeding) [8]. One such enclosure was the European Bison Breeding Center in Smardzewice (central Poland). 

At the end of 2013, 21 European bison were living in Smardzewice. In 2013, the first case of TB was found and the herd was quarantined: no animals could be imported or exported, and visitors were excluded. The probable source of infection was the dam of the positive animal, who was brought from a zoo in which tuberculosis was later found in other species [9]. Since then, following the confirmation of TB by monitoring, the number of individuals began to decrease due to culling, reaching 15 animals in 2014, and seven in 2015. In 2016–2018, only six European bison remained in the herd. In December 2018, the herd was culled [10].

In the years 2013–2018, regular ante-mortem testing for TB was carried out to monitor the epidemiological situation in the herd and to potentially identify animals for euthanasia. A number of ante-mortem methods are used to diagnose TB in European bison, such as serological tests [11], microbiological and molecular methods [8], as well as imaging diagnostics to assess the advance of disease [12]. The present work describes the first evaluation of the use of standard diagnostic methods used in cattle: the tuberculin skin test (TST) and the interferon gamma release assay (IGRA), in European bison. Considering that the European bison is extremely sensitive to mycobacterial infection and its ongoing restitution requires the improvement of ante-mortem diagnostic methods, our findings are of great importance for TB management in this species.

## 2. Results

**Figure 1:**
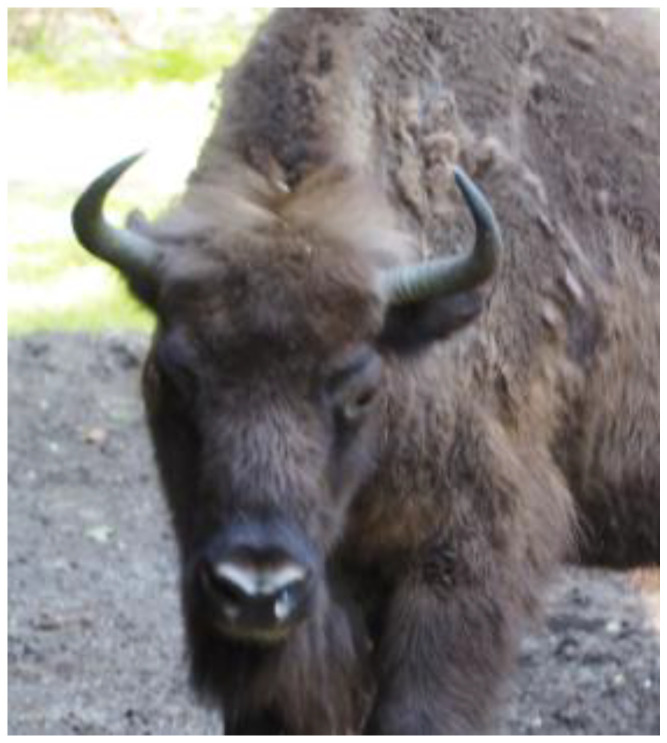
Weak positive result of the intrapalpebral tuberculin skin test (mild swelling of the upper left eyelid).

**Figure 2:**
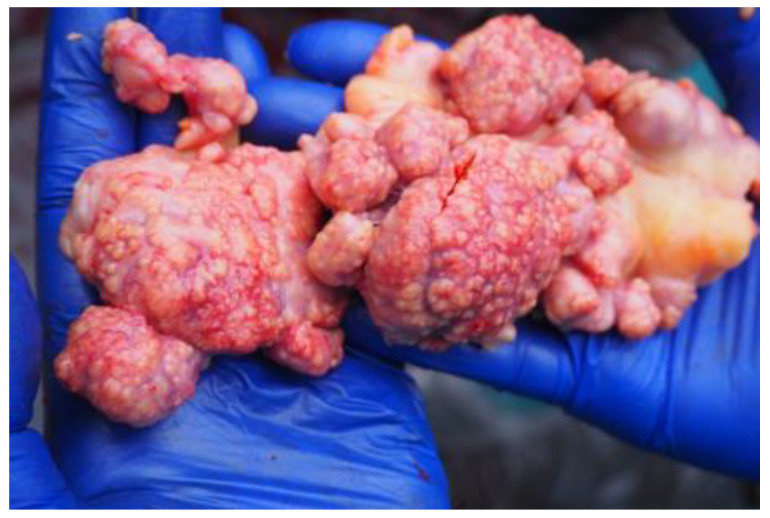
Granulomatous lesions in the lymph nodes in one of the studied European bison.

Clinical signs were limited to exercise intolerance in one animal. In the last test before death or euthanasia in the euthanized European bison, the TST result was positive in one out of eight and weakly positive in three; IGRA was positive in five out of ten (Table 1). In European bison tested with IGRA more than once, the outputs in samples stimulated with bovine PPD increased over time. A mild swelling of the upper eyelid was seen in three animals (weak positive), and a marked swelling of the upper eyelid with purulent discharge was seen in one animal (strong positive) (Figure 1). In the post-mortem examination, tuberculous lesions (Figure 2) and positive culture results were observed in five out of ten individuals. More detailed results are presented in Table 1. The results of the animals subjected to five ante-mortem tests are presented in Table 2. All isolated strains were identified as *Mycobacterium caprae*.

## 3. Discussion

Our findings indicate that, in the European bison, a near threatened species [13], the IGRA is easier to perform and interpret than the palpebral TST. The IGRA showed 100% agreement with the bacteriological culture—gold standard test [14], which suggests that it offers promise as a useful future test. The TST sensitivity was the same, with the note that some of the results were difficult to interpret and were described as “weak positive”. It seems that the best solution in the diagnosis of tuberculosis in European bison would be to use two tests in parallel instead of relying on one method alone. Although our research has some limitations, such as the small sample size, it still achieved some valuable goals, especially considering the protected status of European bison. Interestingly, according to our study, the direct contact is important in the transmission of disease and the age of the animal did not play a role in the severity of disease.

The TST is the primary test used to monitor TB in cattle in Poland and most other countries. However, it should be remembered that the test may demonstrate lower sensitivity in some situations; in addition, false negative or weak positive (hard to interpret) results may be obtained during the early or advanced stages of the disease and in individuals with a reduced immune status, e.g., as a result of infections with viruses with immunosuppressive properties, such as bovine immunodeficiency virus (BIV) or bovine viral diarrhea virus (BVDV) [15]. 

The results of the post-mortem examination suggest that the degree of advancement of the disease had poor correlation to the TST result. The result of the last TST performed was a weak positive in sample period E from animal #7, presenting generalized TB, as well as with animals #6 and #9, which demonstrated tuberculous lesions in the lymph nodes (Table 2). Although some studies have noted a positive correlation between the intensity of skin reactions to bovine tuberculin administration and the extent of pathological lesions found in post-mortem examination [16,17], in our study, it was not confirmed.

Our findings highlight a number of limitations associated with the use of the TST for the diagnosis of TB in European bison. In animals #6, #7, and #9, while the initial TST results were interpreted as positive (swelling and purulent discharge from the left eye), later, test results (C and D) were weakly positive (Table 2). This may indicate reduced sensitivity to tuberculin bPPD; indeed, frequent TST application may lead to a diminished cellular immunological response in subsequent repetitions [18,19]. Such minimal reactions obtained by repeated TST testing have been attributed to increased interleukin-10 (IL-10) and decreased interleukin-1β (IL-1β) production. Increasing IL-10 secretion leads to macrophage inactivation, which, in turn, results in a reduction in the presentation of antigens. In addition, decreased IL-1 β production reduces T lymphocyte activation and, thus, the secretion of proinflammatory cytokines, including INF-γ [20]. Interleukins have been investigated as biomarkers in the assessment of TB staging [21,22] and they may be of value in tests designed for European bison. However, in the conducted study, there was a lack of IGRA responses, which decreased over time (cellular response like TST); one possible explanation was reusing the same site for the TB test. In primates, it is recommended that TST retesting is conducted in the thoracic or abdominal skin [23]. Therefore, cervical TST might be a reasonable option in the case of inclusive results, as caliper measurement allows more objective interpretation; however, the animal would need to be immobilized twice.

In the above-mentioned individuals (#6, #7, #9) the result of the final TST was determined to be a weak positive (sample period E) due to only a slight swelling being observed in the area of the left eye. The evaluation of intra-palpebral TST is extremely subjective, especially in the absence of obvious clinical signs, such as pronounced eyelid edema and ptosis [23]. Additionally, no criteria exist for evaluating the results of the TST in European bison; the veterinarian does not have the possibility of measuring the skinfold with a caliper, as is the case with neck TST in cattle, or by palpation in the case of caudal fold tuberculinization (CFT). Such processes may be used in future TST protocols; however, this would require pharmacological re-immobilization in order to read, which is undesirable in the case of European bison: re-immobilization presents a risk to the health of the animal and requires appropriate consent. As the TST is not validated for European bison, its results should be interpreted with caution. Most importantly, the test results could only be read from a distance, which reduces the sensitivity of the method. Palpebral TST, however, eliminates the need to re-immobilize after 72 hours in order to read the result, as is the case for neck or tail tests. In wild animals, immobilization is always risky and difficult; therefore, a method was chosen that did not require repeated dangerous and costly immobilization procedures. In wild ruminants, the greatest dangers include capture myopathy, bloat, and regurgitation [24,25]. Unfortunately, due to the size of bison and the danger, cattle crushes cannot be used to avoid a second anesthetic. However, the eyelid TST could be made more efficient by photographing the face (both frontal and side) before and after testing, as described in Asiatic lions (*Panthera leo persica*) [26].

The guidelines of the United States Department of Agriculture (USDA) require a diagnosis of TB to be based on the use of CFT [27,28] in American bison (*Bison bison*). CFT offers 50% sensitivity and 88% specificity compared to culture [29]. A study comparing three methods used in American bison (*Bison bison athabascae*) found CFT to demonstrate higher sensitivity and lower specificity compared to fluorescence polarization and rapid lateral flow test [27]. However, this method would be very difficult to perform in European bison because of the necessity to catch the bison in cages twice; as noted above, this is a very difficult and potentially dangerous procedure. Additionally, there are no harvesting facilities in most European bison breeding centers.

Despite the previously mentioned limitations of the TST method, it seems that it may still be of value in European bison in enclosures, especially when positive or inconclusive results are obtained in other indirect tests. It is also very useful to perform comparative tubercularizing to reduce the risk of false-positive results associated with the presence of atypical mycobacteria in the environment [29], particularly since atypical mycobacteria have previously been isolated from European bison [11]. It should be highlighted that using a comparative TST does not eliminate any possible cross-reaction for bovine tuberculin [30]. Moreover, the optimal choice of tuberculin injection site (eyelid, caudal fold, neck) remains to be determined.

In wildlife species, IGRA has the advantage over TST in that it requires only one contact with the animal; it can, therefore, be used for screening in free-ranging herds and animals in enclosures. Even so, the procedure is costly. While it is important that material collection is well organized and that the blood sample is transported as quickly as possible to the laboratory [31], in the present study, the antigens were always added within the time limit specified in the manufacturer’s instructions (up to 30 h from the time of blood collection). 

The results of the IGRA test were found to agree with those of the post-mortem lesion examination and culture, both confirming and excluding TB. It, therefore, appears effective in the ante-mortem diagnosis of TB in European bison (Table 1). However, as in the case of the TST, the IGRA may demonstrate reduced sensitivity with the advancement of the disease [17]. However, no such relationship was observed in the present study; for example, in animal #7, who was found to have generalized tuberculosis in the post-mortem examination, the IGRA result was positive. The output of IGRA was found to increase over time, in line with disease advancement and after injections with bovine PPD for TSTs.

It should be noted that the influence of frequent TST testing on IGRA results is not fully understood [32]. A negative effect (weakening of the cell-type immune response—possible false-negative result) was observed in blood collected for IGRA three days after TST in infected African buffalo [33]. Studies in cattle indicate that a single TST has a positive effect on stimulating the cellular response in IGRA [34]. Other studies state that comparative tubercularizing is not believed to significantly affect the IGRA result [32]. In recent studies conducted in Smardzewice, Poland, the time between the TST and the next IGRA blood sampling was always much longer than the recommended seven days after comparative tuberculinization [17]. This suggests that the TST performed had no effect on the obtained IGRA results.

The IGRA is believed to detect bovine mycobacterial infection at an earlier stage than the TST [17]. However, this was not confirmed in the present studies, where positive reactions were first recorded in the TST and only later in the IGRA. For example, animal #6 was found to have a positive TST in collection B and a positive IGRA in the sample period (Table 2). This may have been due to a booster response elicited by performing the TST. 

One potential limitation of the study is that the tests were used for diagnosing *M. bovis* infection rather than *M. caprae*, two separate species [35]. However, the manual for bovine TB diagnostics of the World Organisation for Animal Health (OIE) [36] indicates that *M. caprae* infection is not substantially different from that of *M. bovis*, and the same tests can be used.

A good example for comparison, and which may represent future research directions to diagnose TB in European bison, is that of African buffalo in South Africa. Studies of the diagnostic possibilities in this group [37] suggest that parallel testing is the most effective approach [38]. Moreover, tests with specific peptides, such as ESAT-6 and CFP-10, may provide greater specificity and a possible correlation with pathological findings, as confirmed in QuantiFERON^®^-TB Gold (QFT) tubes in African buffalo [39]. Moreover, testing based on IP-10 in whole blood yielded satisfying results and, together with IGRA, has started to replace skin testing in this species [40]. This approach should also be tested in European bison, together with direct detection methods, such as qPCR, which have been used in other wildlife species [41]. 

European bison are free-living endangered animals that face many threats, both environmental ones [42] and those associated with infectious diseases [43]. In order to protect them, active species protection measures, including health monitoring, must be carried out. Considering the large number of cases of TB in this species [7], there is a need for a diagnostic algorithm for TB in suspected animals. In addition, being a free-living species, it was difficult to obtain material, especially from positive individuals. We hope our findings will be of value when selecting appropriate TB diagnostic tests in this species in the future. In European bison not suspected of TB, however, we recommend that blood collection and IGRA testing should be performed during any immobilization for any reason, such as transport or putting on telemetry collars. It should be emphasized that, in some countries, the recognized method of testing European bison for TB is the TST in the neck [44,45], and we recommend that these should be supplemented with IGRA test results. 

## 4. Materials and Methods

### 4.1. Material

The study included 10 of the 21 animals (ID = 1–10) (six females and four males) in the studied herd, as these were the only animals remaining when this part of the study began. The studied group ranged in age from 3 to 19 years old. European bison #1–5 were housed together and did not have direct contact with bison #6–10. Three were tested five times (A–E) and one four times (A–C and E), with the last sample (E) collected just before euthanasia (Table 2). The mean interval between collections A–E was nine months. The time between A–B and B–C was nine months, between C–D eleven months, and D–E seven months (median: 10 months, range: 7–11 months). 

Ante-mortem diagnostics were conducted as part of the health monitoring rules, which do not require the consent of the ethics committee under Polish law [46]. The animals were subjected to pharmacological immobilization by remote injection using a Palmer Cap-Chur tranquilization gun. The body weight (BW) of the animals was estimated at 400–650 kg, depending on the individual, and the nutritional status from fair to good. Anesthesia was performed with etorphine hydrochloride (Captivon^®^, 9.8 mg/mL, Wildlife Pharmaceuticals, White River, South Africa) at a dose of 0.005–0.01 mg/1 kg BW and xylazine (Nerfasin^®^, 100 mg/mL, Livisto, Senden, Germany) at a dose of 0.3–0.4 mg/1 kg BW. To revive the animals, the following agents were administered: diprenorphine hydrochloride (Activon^®^, 12 mg/mL, Wildlife Pharmaceuticals, White River, South Africa) in a dose corresponding to the amount of etorphine administered, naloxone hydrochloride (Naloxonum Hydrochloricum WZF 400 µg/mL solution for injection, Polfa, Warsaw, Poland) using 1 ampoule (1 mL) for European bison, and atipamezole hydrochloride (Antisedan^®^, 5 mg/mL, Orion Pharma, Espoo, Finland) at a dose of 0.015–00.2 mg/1 kg BW [47]. The ante-mortem sample collection procedure lasted from 20 to 40 min. Blood was first collected with a 1.2 mm diameter 4 mm long needle in tubes containing lithium heparin (Medlab Products, Raszyn, Poland) from the external jugular vein, and then TST was conducted.

Material was also collected post-mortem during anatomopathological section using the Polish protocol for animal infectious diseases [48]. All procedures were carried out in accordance with the principles of biosecurity. Briefly, an external examination was carried out, followed by an internal examination and a detailed examination of the internal organs. Retropharyngeal and tracheobronchial lymph nodes were collected from each animal. If any lesions were observed, lymph nodes from additional locations and organs were collected. Tissue samples were frozen and stored at −20 °C until microbiological examination. Due to the zoonotic nature of TB in animals, all veterinarians were equipped with aprons, masks, caps, and protective shoes during post-mortem examination. No animal was killed for the purpose of this study. All procedures were conducted in accordance with applicable regulations. Euthanasia was conducted according to the decisions of The Polish Ministry of The Environment by shooting, not for the purpose of this study. 

### 4.2. Tuberculin Skin Test (TST)

**Figure 3:**
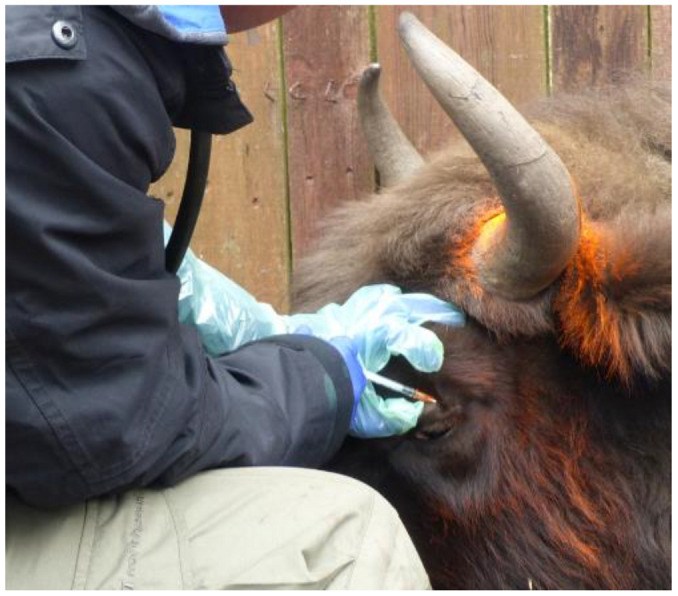
Intradermal administration of bPPD bovine tuberculin to the upper left eyelid in a European bison.

Comparative intrapalpebral TST was performed as described previously [49]. Briefly, the TST was performed with bovine tuberculin purified protein derivative (bPPD) (Bovitubal 28000, Bioveta, Ivanovice na Hané, Czech Republic) in a volume of 0.1 mL (2800 international units of tuberculin) by intradermal injection into the upper left eyelid, approximately 5 mm from the lash line (Figure 3). For comparative TST, avian PPD (aPPD) (Avitubal 28000, Bioveta, Ivanovice na Hané, Czech Republic) was applied in the same way to the upper right eyelid. The correctness of the procedure was confirmed by the presence of a small spherical bleb with a diameter of about 8 mm at the injection site, as revealed by palpation.

The results were assessed after 72 ± 2 h. For this purpose, the animals were not immobilized. The nature and size of the reaction was assessed from a distance of about two meters with the use of binoculars. The image of reactions was recorded with a digital camera. Based on the protocol used in primates [23], interpretation of the test was considered positive if a swelling or significant discharge appeared from the left eye (bovine PPD) and negative if no such sign was observed on the left eye. If both eyelids swelled (left—bovine PPD, and right—avian PPD) the result was inconclusive. If the animal was previously positive for tuberculosis, any slight reaction on the left eye was interpreted as a “weak positive”.

### 4.3. Interferon Gamma Release Assay (IGRA)

The IGRA was performed no later than 24 h after blood collection. After collection, the blood was stored in at a temperature of 18–22 °C until analysis; this was facilitated in field conditions by the use of an incubator equipped with a battery. The IGRA was performed using the commercial Bovigam^®^ TB Kit (Prionics, Schlieren, Switzerland) according to the manufacturer’s instructions. Briefly, whole blood was stimulated with the antigens bPPD 3000 (Prionics, Switzerland) and aPPD 3000 (Prionics, Schlieren, Switzerland). PBS was used as a control. A total of 150 µL of the appropriate antigen diluted in the RPMI-1640 medium (Sigma-Aldrich, Saint Louis, MO, USA) was added to 1.5 mL of whole blood. After incubation (37 °C, 24 h), 500 µL of plasma was collected from each tube and the INF-γ concentration was determined by ELISA. Readings were performed using an EPOCH spectrophotometer (BioTek Instruments Inc., Winooski, VT, USA).

### 4.4. Microbiological and Molecular Examination 

The culture was performed according to the standard procedure used at the National Veterinary Research Institute (NVRI) in Pulawy (Poland). Briefly, the collected material was decontaminated with a 5% solution of oxalic acid (Sigma-Aldrich, Saint Louis, MO, USA). Homogenization was performed in a stomacher (MiniMix, Interscience, France) for three minutes (1500 g, 10 min). The supernatant was removed and the pellet was washed twice with sterile physiological sodium chloride solution (Polfa, Lublin, Poland).

The obtained pellet was seeded on two solid media: Stonebrink (Becton Dickinson, Franklin Lakes, NJ, USA) and Löwenstein–Jensen (Becton Dickinson, Franklin Lakes, NJ, USA). The media were incubated at 37 °C. Growth was assessed every seven days for 12 weeks.

The DNA was isolated from the cultures and the genotype determined as described previously [1]. Briefly, DNA isolation was performed using a Genolyse isolation kit (Hain Lifescience, Nehren, Germany). Strains were determined using the GenoType^®^MTBCassay (Hain Lifescience, Nehren, Germany).

## 5. Conclusions

Our findings indicate that the IGRA is an easier to interpret diagnostic test for TB in European bison than the intrapalpebral comparative TST. Numerous implementations of the TST in field work have also been demonstrated. We recommend the IGRA test be combined with TST for detecting cellular immune responses in the course of TB in European bison. However, it should be borne in mind that our tests were carried out on a small number of animals and further studies are needed to more precisely determine their sensitivity and specificity for European bison. 

## Figures and Tables

**Table 1:** Results of ante-mortem (last before death or euthanasia) and post-mortem testing of tuberculosis of tested European bison.

Animal ID (Age in Years)	TST	IGRA	Lesions	Culture
1 (3)	Not tested	−		
2 (17)	+	+	caseous necrosis in retropharyngeal and mesenteric lymph nodes	+
3 (3)	−	−	−	
4 (5)	−	−	−	
5 (7)	−	−	−	
6 (6)	(±)	+	caseous necrosis in retropharyngeal, tracheobronchial, and mediastinal lymph nodes	+
7 (10)	(±)	+	generalized tuberculous lesions	+
8 (7)	not tested	+	caseous necrosis in retropharyngeal, tracheobronchial, and mediastinal lymph nodes, enlarged duodenal and mesenteric lymph nodes	+
9 (6)	(±)	+	caseous necrosis in retropharyngeal, tracheobronchial, and mediastinal lymph nodes	+
10 (19)	−	−	−	

**Table 2:** Results in European bison tested more than once.

Animal ID	Sample Period	TST	IGRA	Culture
6	A	−	−	+
	B	+	−	
	C	−	−	
	D	−	+	
	E	±	+	
7	A	+	−	+
	B	+	−	
	C	−	−	
	D	−	±	
	E	±	+	
8	A	−	−	+
	B	−	−	
	C	−	−	
	D	Not tested	Not tested	
	E	Not tested	+	
9	A	−	+	+
	B	+	−	
	C	−	−	
	D	−	+	
	E	±	+	

## Data Availability

Data are available in the Department of Food Hygiene and Public Health Protection, Institute of Veterinary Medicine, Warsaw University of Life Sciences (SGGW), Poland.
